# An Analysis of Body Language of Patients Using Artificial Intelligence

**DOI:** 10.3390/healthcare10122504

**Published:** 2022-12-10

**Authors:** Rawad Abdulghafor, Abdelrahman Abdelmohsen, Sherzod Turaev, Mohammed A. H. Ali, Sharyar Wani

**Affiliations:** 1 Department of Computer Science, Faculty of Information and Communication Technology, International Islamic University Malaysia, Kuala Lumpur 53100, Malaysia; 2 Department of Computer Science and Software Engineering, College of Information Technology, United Arab Emirates University, Al Ain 15551, United Arab Emirates; 3 Department of Mechanical Engineering, Faculty of Engineering, University of Malaya, Kuala Lumpur 50603, Malaysia

**Keywords:** body language, pandemic, epidemic, body language analysis, AI, gesture recognition, fall detection, anomaly detection, COVID-19, neocoronal pneumonia

## Abstract

In recent decades, epidemic and pandemic illnesses have grown prevalent and are a regular source of concern throughout the world. The extent to which the globe has been affected by the COVID-19 epidemic is well documented. Smart technology is now widely used in medical applications, with the automated detection of status and feelings becoming a significant study area. As a result, a variety of studies have begun to focus on the automated detection of symptoms in individuals infected with a pandemic or epidemic disease by studying their body language. The recognition and interpretation of arm and leg motions, facial recognition, and body postures is still a developing field, and there is a dearth of comprehensive studies that might aid in illness diagnosis utilizing artificial intelligence techniques and technologies. This literature review is a meta review of past papers that utilized AI for body language classification through full-body tracking or facial expressions detection for various tasks such as fall detection and COVID-19 detection, it looks at different methods proposed by each paper, their significance and their results.

## 1. Introduction

One of the languages of communication is body language. Languages are divided into two categories: verbal and nonverbal. Body language is a type of nonverbal communication in which the body’s movements and actions are utilized instead of words to communicate and transmit information. According to [1,2], nonverbal cues such as gestures, body posture, eye movement, facial expressions, touch, and personal space utilization are all examples of body language.

Body language analysis is also necessary to avoid misunderstandings about the meanings and objectives of a single movement that has several meanings. gaze direction; iris extension; hand and leg position; sitting, walking, standing, or lying manner; body posture; and movement are all examples of how a person’s inner state is portrayed. Hands are arguably the richest wellspring of body language information after the face [3]. For example, one may tell if a person is honest (by turning the hands inside towards the interlocutor) or disingenuous (by turning the hands outside towards the interlocutor) (hiding hands behind the back). During a conversation, using open-handed gestures might convey the image of a more trustworthy individual, a tactic that is frequently employed in discussions and political conversations. It has been demonstrated that persons who make open-handed gestures are liked [4]. The posture of one’s head may also indicate a lot about one’s emotional state: people are more likely to talk more when the listener supports them by nodding. The rate at which you nod might indicate whether you have patience or not. The head is still at the front of the speaker in a neutral stance. When a person’s chin is elevated, it might indicate dominance or even arrogance. Revealing the neck could be interpreted as a gesture of surrender.

In the last few years, automatic body language analysis has gained popularity. This is due in part to the large number of application domains for this technology, which range from any type of human–computer interaction scenario (e.g., affective robotics [5]) to security (e.g., video surveillance [6]), to e-Health (e.g., therapy [7] or automated diagnosis [8]) are examples of e-Health, as are language/communication, e.g., sign language recognition [9]), and amusement (e.g., interactive gaming [10]). As a result, we can find research papers on a variety of topics related to human behavior analysis, such as action/gesture recognition [11,12], social interaction modeling [13,14], facial emotion analysis [15], and personality trait identification [16], to name a few. Ray Birdwhistell conducted research on using body language for emotional identification and discovered that the final message of a speech is altered only 35 percent by the actual words and 65 percent by nonverbal signals [17]. In addition, according to psychological study, facial expression sends 55 percent of total information and intonation expresses 38 percent in communication [4].

We will provide a new thorough survey in this study to help develop research in this area. First, we provide a description and explanation of the many sorts of gestures, as well as an argument for the necessity of instinctive body language detection in determining people’s moods and sentiments. Then we look at broad studies in the realm of body language processing. After that, we concentrate on the health care body language analysis study. Furthermore, we will define the automated recognition frame for numerous body language characteristics using artificial intelligence. Furthermore, we will describe an automated gesture recognition model that aids in the better identification of epidemic and pandemic illness external signs.

## 2. Body Language Analysis

### 2.1. Overview of Body Language Analysis

Body language interpretations fluctuate from nation to country and from culture to culture. There is substantial debate about whether body language can be considered a universal language for all humans. Some academics believe that most of the interpersonal communication is based on physical symbols or gestures, because the interplay of body language enhances rapid information transfer and comprehension [18]. 

Body language analysis is also necessary to avoid misunderstandings about the meanings and objectives of a single movement that has several meanings. A person’s expressive movement, for example, may be caused by a physical limitation or a compulsive movement rather than being deliberate. Furthermore, one person’s bodily movement may not signify the same thing to another. For example, itching may cause a person to massage her eyes rather than weariness. Because of their societal peculiarities, other cultures also require thorough examination. There are certain common body language motions, but there are also movements unique to each culture. This varies depending on the nation, area, and even social category. In this chapter of this study, we will discuss the various aspects of body language analysis and will explain this below.

### 2.2. Body Language Analysis in Communication

In research from [19], body language is a kind of nonverbal communication. Humans nearly exclusively transmit and interpret such messages subconsciously. Body language may provide information about a person’s mood or mental condition. Aggression, concentration, boredom, relaxed mood, joy, amusement, and drunkenness are just a few of the messages it might convey. Body language is a science that influences all aspects of our lives. Body language is a technique through which a person may not only learn about other people by observing their body motions but also improve himself properly and become a successful person. Body language is a form of art that allows a person to acquire a new level of fame.

If language is a way of social connection, then body language is unquestionably a reflection of personality development. It can allow for reading other people’s minds, allowing a person to effortlessly mold himself to fit the thinking of others and make decisions for effective and impactful planning. The person’s mental mood, physical fitness, and physical ability are all expressed through body language. It allows you to have a deeper knowledge of individuals and their motives. It builds a stronger bond than a lengthy discussion or dispute. Reading body language is crucial for appropriate social interaction and nonverbal communication.

In human social contact, nonverbal communication is very significant. Every speaking act we perform is accompanied by our body language, and even if we do not talk, our nonverbal behavior continually communicates information that might be relevant. As a result, the following research [20] seeks to provide a summary of many nonverbal communication components. Nonverbal communication is usually characterized as the opposite of verbal communication: any occurrences with a communicative value that are not part of verbal communication are grouped under the umbrella term nonverbal communication, as well as auditory factors such as speaking styles and speech quality. On the one hand, paralinguistic (i.e., vocal) phenomena such as individual voice characteristics, speech melody, temporal features, articulation forms, and side noise can be found.

**Figure 1:**
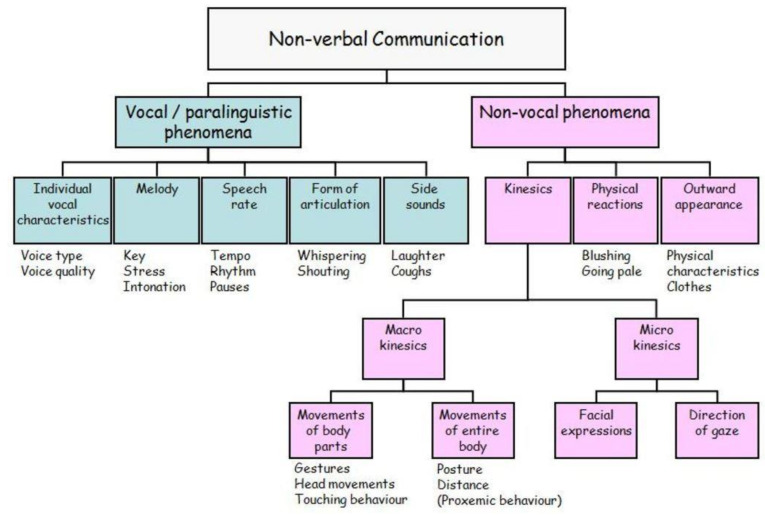
Overview of the main forms of nonverbal communication. The figure has been taken from [20].

Nonvocal phenomena in conversation, on the other hand, include a speaker’s exterior traits, bodily reactions, and a variety of kinesics phenomena that can be split into macro-kinesics and micro-kinesics phenomena. Figure 1 depicts a comprehensive review of the many types of nonverbal communication.

In this study from [21], nonverbal conduct encompasses all forms of communication other than speaking. The term “communication” refers to the act of sending and receiving messages. Even though language use is a uniquely human trait, differing perspectives revolve around nonverbal behaviors and the current context. We employ body language without realizing it, as well as see and understand the body language of others. Nonverbal conduct is divided into three categories: verbal–vocal, nonverbal vocal, and nonverbal nonvocal. The link between verbal and nonverbal conduct is demonstrated through several gestures. Nonverbal events have a crucial role in the structure and occurrence of interpersonal communication, as well as the interaction’s movement-to-movement control. Nonverbal cues such as hierarchy and priority among communicators, signaling the flow of interaction, and giving meta-communication and feedback contribute to governing the system.

As shown in [22], body language is one of the most crucial aspects of communication. Communication that cannot be resolved due to body language stays unfinished. Our physical appearance also has a significant impact on how well we deliver our message. Our thoughts, expressions, postures, and gestures all have a significant impact on the weight of meaning and emotion carried by our phrases and words. Understanding and conveying emotions and thoughts rely heavily on body language. It is important for the proper expression and comprehension of messages during the communication process. It also promotes oral communication and establishes communication integrity. Body language accounts for 55% of how we impress people when speaking, words account for 7%, and discourse accounts for 38%.

It is critical to concentrate on this distinction if you want to be an effective speaker. Because body language is swiftly registered in the subconscious, the audience focuses on it. Even if the audience does not comprehend the spoken language, the audience may grasp the message through body language.

### 2.3. Body Language in Public Speaking

Although our face is the indicator of our thinking, we cannot deny that words are also quite powerful. We may look to the French and Russian Revolutions for instances of great speeches delivered by leaders. However, we cannot afford to overlook the reality that actions speak louder than words, i.e., body language is more potent than words. We use words to disguise our emotions and sentiments much of the time, but our body language makes them quite evident. Our formal and professional lives are completely reliant on nonverbal communication, which we engage in through our behaviors and body language. People in the office do not speak much yet transmit everything through their body language. Whenever they communicate, they consciously or unconsciously use their body language more than their words. In any conversation, body language is important. The image of Lord Krishna speaking to Arjuna on the fields of Kurukshetra will be read, described, and analyzed in this research study [23]. It will discuss the significance of body language when speaking in public.

### 2.4. Body Language Analysis in Teaching

The main goal of the study [24], was to assess the influence of teachers’ nonverbal communication on teaching success based on the findings of research on the link between teaching quality and teachers’ nonverbal communication and its impact on teaching success. The study results demonstrated that there was a substantial link between the quality, quantity, and technique of nonverbal communication used by instructors when instructing. According to the study’s findings of the research evaluated, the more teachers using verbal and nonverbal communication, the more effective their instruction and the academic achievement of their pupils were.

According to other research, why do certain teachers exude a mystical charisma and charm that sets them different from their colleagues? The Classroom X-Factor investigates the idea of possessing what the public has come to refer to as the “X-Factor” from the perspective of the teacher, providing unique insights into the use of nonverbal communication in the classroom. This study shows how both trainee and practicing teachers may find their own X-Factor to assist shift their perspectives and perceptions of themselves during the live act of teaching, using classroom and curricular provided examples. It also shows how instructors may change the way they engage with their students while simultaneously providing them with significant and powerful learning opportunities. Teachers may generate their own X-Factor by adopting easy strategies derived from psychology and cognitive science, and therefore boost their satisfaction and efficacy as professionals. Facial and vocal expression, gesture and body language, eye contact and smiling, teacher apparel, color and the utilization of space, nonverbal communication, and educational approaches are among the tactics outlined. Furthermore, the study includes a part with fictitious anecdotes that serve to contextualize the facts presented throughout the text [25].

### 2.5. Body Language Analysis in Sport

The literature reviewed shows that nonverbal behavior (NVB) changes as a result of situational variables, because either a person shows a nonverbal response to provoking internally and externally circumstances (as is theorized for just some basic emotions conveyed in the face) or because a person intentionally desires to convey certain information nonverbal cues to observers in a given situation. Certain NVBs have been demonstrated to have a range of consequences on later interpersonal results, including cognition, emotion, and behavior, when they are displayed and seen (e.g., [26] for reviews). 

### 2.6. Body Language Analysis in Leadership

The authors of [27] examined the possibility of gender disparities in leaders’ nonverbal actions, as well as the impact these differences may have on their relative effectiveness. Nonverbal communication may reveal a leader’s emotions and increase followers’ involvement. Once the leader is aware of his or her gestures and body motions, he or she may compare them to those of more effective leaders. On certain levels, gender inequalities in nonverbal behavior occur. Women are linked to transformative traits such as compassion, love, and concern for others. Men, on the other hand, relate to traits such as aggressiveness, dominance, and mastery.

This demonstrated that productive women do not always exhibit the same nonverbal behaviors as effective males. Nonverbal hesitations, tag questions, hedges, and intensifiers are more likely to be used by fluent speakers. This suggests that leaders who shake their heads are more likely to exhibit higher counts of speaker fluency behaviors. It is also not tied to gender in any way. Another intriguing finding is that the head movement of nodding is linked to the behaviors of upper grin, broad smile, and leaning forward. This demonstrates that these affirming, good behaviors are linked in a major way. Furthermore, the observed leaders’ speech fluency is substantially connected with their head movement shaking.

### 2.7. Body Language Analysis in Culture

In [28], the authors discussed a range of body languages used in many civilizations throughout the world. The meanings that may be conveyed through body language are numerous. The following is an example: People from all cultures use the same body language, such as staring and eye control, facial emotions, gestures, and body movements, to communicate their common sense. Distinct cultures have different ways of communicating non-verbally, and varied people have different ways of expressing themselves via gestures. Nonverbal communication, in the same way as traffic, has a purpose and follows a set of norms to ensure that it flows smoothly among people from many diverse cultures.

On the other hand, cultures can use the same body language to communicate diverse meanings. There are three sides to it:

- Eye contact differs by culture.
- Other nonverbal signals vary by culture.
- The right distance between two individuals reveals their distinct attitudes from different civilizations.

Our culture is as much about body language as it is about verbal discourse. Learning the various basic norms of body language in other cultures might help us better understand one other. People from many cultures are able to converse with one another. However, cultural exchanges and cultural shocks caused by our body language are becoming increasingly harsh and unavoidable.

As a result, while communicating in a certain language, it is best to utilize the nonverbal behavior that corresponds to that language. When a person is fully bilingual, he changes his body language at the same time as he changes his language. This facilitates and improves communication.

Lingua franca is a linguistic bridge that connects two persons who speak different native languages.

In this regard, it has been determined in [29] that we communicate with our vocal organs and that our bodies’ body language can be a lingua franca for multilingual interlocutory.

The findings indicate that the listener was attempting to comprehend the speaker’s gesture. Because the speaker cannot speak English fluently, he was having difficulty achieving precise diction. The speaker ultimately succeeded in expressing his views with gestures towards the end of the video. Furthermore, the interlocutors were involved in the delivery and reception of suggested meaning via gestures and body language. Even though a lingua franca (e.g., English) already existed, body language adds significance to the message.

Furthermore, according to the data collected in this study, the Korean model and a client had a tumultuous history while shooting certain photoshoots. The customer was not pleased with the model’s attitude, which he felt insulted him. Nonetheless, the Korean model apologized in a traditional Korean manner by kneeling to the customer and the judges.

The judges and the client were both impressed by her formal and courteous demeanor. To finish the analysis, this research employed multimodal transcription analysis with Jefferson and Modada transcript notation, as well as YouTube data clips. Some mistakes may continue, which might be an excellent starting point for additional study in the fields of lingua franca and body language to gain a more comprehensive understanding.

### 2.8. Body Language in Body Motions

Both cognitive-based social interactions and emotion-based nonverbal communication rely heavily on body movements and words. Nodding, head position, hand gestures, eye movements, facial expressions, and upper/lower-body posture, as well as speaking, are recognized to communicate emotion and purpose in human communication.

#### 2.8.1. Facial Expressions

According to new research, facial expressions are changes in the appearance of the face caused by the movement of facial muscles. it is a nonverbal communication route. Emotional facial expressions are both symptoms and communication cues of an underlying emotional state. People seldom convey their feelings by using characteristic expressions connected with certain emotions that are also widely recognized across countries and settings. Furthermore, environmental circumstances have a significant impact on both the expression and detection of emotional responses by observers [30].

In recent years, as [31] notes, there has been a surge in interest in both emotions and their regulation, notably in the neurosciences and, more specifically, in psychiatry. Researchers have attempted to uncover patterns of expression in experimental investigations analyzing facial expressions. There is a large amount of data accessible; some of it has been validated, while others have been refuted, depending on the emotion studied and the method employed to assess it. Interpreting data that have not always been completely proven and are based on Paul Ekman’s hypothesis of six main types of expression is a key issue (happiness, anger, disgust, fear, sadness, and surprise).

The sense of happiness, with its expressive element of the “smile,” is the only one of Ekman’s “basic emotions” that is observably linked to the underlying physiological and facial pattern of expression. Regarding Ekman’s other basic patterns of expression, there is much scholarly debate. A better understanding of how emotions are regulated and how the dynamics of emotional facial expression may be described could lead to more basic research in a social situation. Even more crucially, it has the potential to increase knowledge of the interaction and social repercussions of emotional expression deficiencies in people with mental illness, as well as a therapeutic intervention. Innovative study in the realm of emotional facial expression might give thorough solutions to unanswered issues in the field of emotion research.

#### 2.8.2. Gestures

Gestures are generally hand gestures (but they can also include head and facial movements) that serve two purposes: to illustrate speech and to transmit verbal meaning. Gestures are fascinating because they represent a sort of cognitive science; that is, they are motions that express an idea or a mental process [32].

Whenever a person is pondering what to say, gesturing relieves the cognitive burden. When people have been given a memory job while also explaining how and where to solve a math issue, for example, they recall more objects if they use gestures while describing the arithmetic. When counting objects, being able to point allows for higher precision and speed; when people are not permitted to tell, even nodding allows for greater precision [33]. Gestures aid in the smoothing of interactions and the facilitation of some components of memory. As a result, gestures can provide valuable insight into speakers’ states of mind and mental representations. Gestures may be divided into two types: those that occur in conjunction with speech and those that exist independently of speech [26].

## 3. Body Language Analysis and AI

### 3.1. Overview

In face-to-face talks, humans have demonstrated a remarkable capacity to infer emotions, and much of this inference is based on body language. Touching one’s nose conveys incredulity, whereas holding one’s head in the hands expresses upset among individuals of comparable cultures. Understanding the meaning of body language appears to be a natural talent for humans. In [34], the authors presented a two-stage system that forecast emotions related to body language with normal RGB video inputs to assist robots to develop comparable skills. The programmed guessed body language using input movies based on approximated human positions in the first step. After that, the expected body language was transmitted into the second step, which interpreted emotions. 

Automated emotion identification based on body language is beneficial in a variety of applications, including health care, internet chatting, and computer-mediated communications [35]. Even though automated body language and emotions identification algorithms are used in a variety of applications, the body language and emotions of interest vary. Online chatting systems, for example, are focused on detecting people’s emotions, i.e., if they are happy or unhappy, whereas health care applications are concerned with spotting possible indicators of mental diseases such as depression or severe anxiety. Because a certain emotion can only be expressed through the associated body language, many applications necessitate the annotation of various body language and emotions.

### 3.2. Recognition of Facial Expressions

Facial expressions (FE) are important affect signaling systems that provide information about a person’s emotional state. They form a basic communication mechanism between people in social circumstances, along with voice, language, hands, and body position. AFER (automated FE recognition) is a multidisciplinary field that straddles behavioral science, neuroscience, and artificial intelligence.

**Figure 2:**
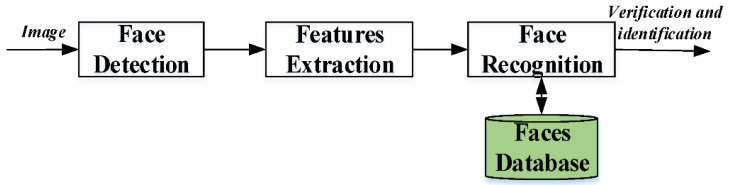
Face recognition structure. The figure has been taken from [39].

Face recognition is a prominent and well-established topic in computer vision. Deep face recognition has advanced significantly in recent years, thanks to the rapid development of machine learning models and large-scale datasets. It is now widely employed in a variety of real-world applications. An end-to-end deep face recognition system produces the face feature for the recognition given a natural picture or video frame as input [36]. Face detection, feature extraction, and face recognition (seen in Figure 2) are the three main phases in developing a strong face recognition system [37,38]. The face detection stage is utilized to recognize and locate the system’s human face picture. The feature extraction stage is used to extract feature vectors for every human face that was found in the previous step. Finally, this face recognition stage compares the retrieved characteristics from the human face with all template face databases to determine the human face identification.

#### 3.2.1. Face Detection

The face recognition system starts with the identification of human faces in each picture. The goal of this phase is to see if there are any human faces in the supplied image. Face detection might be hampered by fluctuations in lighting and facial expression. Pre-processing activities are carried out to enable the creation of a more robust face recognition system. Many approaches, such as in [40] and the histogram of oriented gradient (HOG) [41], are utilized to identify and locate the human face picture. Face detection may also be utilized for video and picture categorization, among other things.

#### 3.2.2. Feature Extraction

The major purpose of this phase is to extract the characteristics of the face photos that were discovered in the detection stage. This stage defines a face using a “signature,” which is a set of characteristic vectors that characterize the major aspects of the face picture, such as the mouth, nose, and eyes, as well as their geometric distribution [42]. Each face has a unique structure, size, and form that allows it to be recognized. To recognize the face using size and distance, some ways involve extracting the contour of the lips, eyes, or nose [37]. To extract facial characteristics, approaches such as HOG [43], independent component analysis (ICA), linear discriminant analysis (LDA) [44], and scale-invariant feature transform (SIFT) [38], and local binary pattern (LBP) [42] are commonly utilized.

#### 3.2.3. Face Recognition

This phase compares the features derived from the backdrop in the feature extraction stage to recognized faces recorded in a database. Face recognition may be used for two different purposes: identification and verification. A test face is compared with a set of faces during the identification process to discover the most likely match. To determine the approval or rejection decision, a test face is compared with a known face in the database during the identification process [45]. This challenge has been successfully addressed by correlation filters (CFs) [46], convolutional neural networks (CNN) [47], and k-nearest neighbor (K-NN) [48].

### 3.3. Face Recognition Techniques

Considering the data that have been reported thus far, these authors [44] believed that three techniques stand out as particularly promising for future development in this area: (i) the development of 3D face recognition, (ii) the use of multimodal fusion techniques of complementary data types, particularly those based on visible and near-infrared images, and (iii) the use of deep learning methods.

#### 3.3.1. D Facial Recognition

Due to the 3D structure of the face, some characteristics are lost in 2D image-based approaches. Two key unsolved issues in 2D face recognition are lighting and position variability. The scientific community has recently focused on 3D facial recognition to tackle unsolved challenges in 2D face recognition and obtain considerably greater accuracy by assessing the geometry of hard features on the face. As a result, various contemporary techniques based on 3D datasets in [49,50] have been created.

#### 3.3.2. Multimodal Facial Recognition

Sensors with the demonstrated capacity to capture not only 2D texture information but also face shape, that is, three-dimensional information, have been created in recent years. As a result, several recent studies have combined the two forms of 2D and 3D information to take use of each and create a hybrid system that increases recognition as a single modality [51].

#### 3.3.3. Deep Learning Facial Recognition

DL is a wide notion with no precise definition; nonetheless, researchers [52,53] have agreed that DL refers to a collection of algorithms that aim to model high-level abstractions by modeling several processing levels. This field of study, which dates to the 1980s, is a branch of autonomous learning in which algorithms are employed to create deep neural networks (DNN) that are more accurate than traditional procedures. Recently, progress has been made to the point that DL outperforms humans in several tasks, such as object recognition in photos.

### 3.4. Recognition of Gestures

We reviewed contemporary deep-learning-based algorithms for gesture identification in videos in this part, which are primarily driven by the fields of human–computer, machine–human, and robot interaction.

#### 3.4.1. Convolutional Neural Networks in 2D

Applying 2D CNNs to individual frames and afterward averaging the result for categorization is the first way that immediately springs to mind for identifying a sequence of pictures. In [54], it has been described a CNN machine learning framework for human posture estimation and constructs a spatial component that tries to make joint predictions by considering the locations of related joints. They train numerous convents to perform binary body-part categorization independently (i.e., presence or absence of that body part). These nets are applied to overlapping portions of the input as sliding windows, resulting in smaller networks with greater performance. However. a CNN-based mega model for human posture estimation has been presented in [55]. The authors extract characteristics from the input picture using a CNN. These characteristics are subsequently fed into joint point regression and body part identification tasks. For gesture recognition gesture identification (fingers spelling of ASL) using depth pictures, Kang et al. (2015) use a CNN to extract the features from the fully connected layers. Moreover, a deep learning model for estimating hand posture that uses both unlabeled and synthesized created data is offered in [56]. The key to the developed framework is that instead of embedding organization in the model architecture, the authors incorporate information about the structure into the training approach by segmenting hands into portions. For identifying 24 American Sign Language (ASL) hand movements, CNN and stacked de-noising autoencoder (SDAE) were employed in [57]. A multiview system for point cloud hand posture identification has been showed in [58]. It has been created view picture by projecting the hand point cloud onto several view planes and then feature extraction from these views using CNN. A CNN that uses a GMM-skin detector to recognize hands and then align them to the major axes has been presented in [59]. After that, they used a CNN with pooling and sampling layers, as well as a typical feed-forward NN as a classifier.

Meanwhile, a CNN that retrieves 3D joints based on synthetic training examples for hand position prediction has been presented in [60]. A neural network turns its output of the convolution layer to heat maps (one for each joint) on top of the final layer, displaying the likelihood for each joint. An optimization problem is used to recover poses from a series of heatmaps.

#### 3.4.2. Features That Are Dependent on Motion

Gesture recognition has been widely utilized using neural networks and CNNs based on body posture and hand estimation as well as motion data. To achieve better results, temporal data must be incorporated into the models rather than geographical data. Two-stream (spatiotemporal) CNNs to learn from a set of training gestures for gesture style detection in biometrics have been studied in [61]. The spatial network is fed with raw depth data, while the temporal network is fed with optical flow. However, color and motion information to estimate articulated human position in videos were used in [62]. With an RGB picture and a collection of motion characteristics as input data, the authors present a convolutional network (ConvNet) framework for predicting the 2D position of human joints in the video. The perspective projections of the 3D speed of sliding surfaces are one of the motion characteristics employed in this technique. For gesture identification from depth data, three forms of dynamic-depth image (DDI), dynamic-depth normal image (DDNI), and dynamic-depth motion normal image (DDMNI), as with the input data of 2D networks, were employed in [54]. The authors used bidirectional rank pooling to create these dynamic pictures from a series of depth photos. These representations are capable of successfully capturing spatiotemporal data. A comparable concept of gesture recognition in continual depth video is proposed in [41]. They determine the utter and total depth difference between the current frame and the beginning frame for every gesture segment, which is a kind of motion characteristic as the input data of a deep learning network, and then they begin building an improved depth motion map (IDMM) by calculating the utter and total depth difference between the current frame and the beginning frame for each gesture segment; this serves as a kind of motion characteristic as the input data of a deep learning network.

#### 3.4.3. Convolutional Neural Networks in 3D

Many 3D CNNs for gesture recognition have been presented by [3,49,63], where a three-dimensional convolutional neural network (CNN) for recognizing driver hand gestures based on depth and intensity data has been presented in [3]. For the final forecast, the authors use data from several spatial scales. It also makes use of spatiotemporal data enrichment for even more effective training and to avoid overfitting. However, a recurrent mechanism to the 3D CNN to recognize and classify dynamic hand movements has been added in [55]. A 3D CNN is used to extract spatiotemporal features, a recurrent layer is used for global temporal modeling, and a SoftMax layer is used to forecast class-conditional gesture probabilities. Continuously, a 3D CNN for sign language identification that extracts discriminative spatiotemporal characteristics from a raw video stream has been presented in [63]. (RGB-D and Skeleton data) of streaming video, containing color information, depth clue, and body joint locations, are utilized as input to the 3D CNN to improve the performance has been offered in [64]. By merging depth and RGB video, a 3D CNN model for large-scale gesture detection. In a similar vein, an end-to-end 3D CNN based on the model of [65] and uses it for large-scale gesture detection has been pointed in [66], the wide range of use cases of CNNs for various gesture recognition tasks across the years proves their effectiveness in such tasks, the presence of an extra dimension makes 3D CNNs unique in that the third dimension can be mapped to a time dimension to process videos or a depth dimension to acquire more useful data for a task as seen in [67]. Previous literature support this finding by indicating that combining 3D-based CNNs with temporal models such as an RNN yields desirable results and allows the usage of continuous streams such as videos, currently, CNNs are widely utilized for 2D and 3D based image and gesture recognition and detection tasks.

#### 3.4.4. RNN and LSTM Models for Temporal Deep Learning

Interestingly, despite being a promising study area, periodic deep learning models have still not been frequently employed for gesture identification. In [68], it has been offered a multimodal (depth, skeleton, and voice) gesture recognition system based on RNN, which we are aware of. Each modality is initially processed in small spatiotemporal blocks, wherein discriminative data-specific characteristics are either manually retrieved or learned. After that, RNN is used to simulate large-scale temporal relationships, data fusion, and gesture categorization. Furthermore, in [69] it has been studied a multi-stream RNN for large-scale gesture detection. [70] proposes a convolutional long short-term memory recurrent neural network (CNNLSTM) capable of learning gestures of various lengths and complexity. Faced with the same challenge [71], it has been suggested MRNN, a multi-stream model that combines RNN capabilities with LSTM cells to help handle variable-length gestures. However, in [51] sequentially supervised long short-term memory (SS-LSTM) has been suggested; wherein auxiliary information is employed as sequential supervision at each time step instead of providing a class label to the output layer of RNNs. To identify sample frames from the video sequence and categorize the gesture, the authors in [49] have been employed a deep learning architecture. To build the tiled binary pattern, they use a tiled picture formed by sampling the whole movie as that of the input of a reconvened. The trained long-term recurring convolution network then receives these representative frames as input. However, it has been presented in [71] an EM-based approach for poor supervision that integrates CNNs with hidden Markov-Models (HMMs).

## 4. Body Language Analysis of Patients and AI

### 4.1. Overview

Different artificial intelligence (AI) methods and techniques have been used in analyzing the body language of the patients. Machine learning methods showed a high level of flexibility to a variety of pharmacological conditions. We briefly discuss some studies held so far in this area. 

### 4.2. Facial Recognition

More specifically, focusing on facial recognition, a pimple system called the facial action coding system (FACS) was introduced in [71] to analyze facial muscles and thus identify different emotions. The proposed system automatically tracks faces using video and extracts geometric shapes for facial features. The study was conducted on eight patients with schizophrenia, and the study collected dynamic information on facial muscle movements by going through the specifics of the automated FACS system and how it may be used for video analysis. There are three steps to it. The first stage (image processing) explains how face photos are processed for feature extraction automatically. Next is action unit detection, which explains how we train and evaluate action unit classes. The process finishes (application to video analysis) by demonstrating how to utilize classifiers to analyze movies to gather qualitative and quantitative data on affective problems in neuropsychiatric patients. This study showed the possibility of identifying engineering measurements for individual faces and determining their exact differences for recognition purposes. As a result, Controls 3, 2, and 4 patients were quite expressive, according to the automated evaluation, but patients 1, 2, and 4 were relatively flat. Control 1 and patient 3 were both in the middle of the spectrum. Patients 4 and 3 had the highest levels of inappropriate expressiveness, whereas patients 1 and controls 1–4 had moderate levels.

Three methods were used in [31] to measure facial expression to determine emotions and identify persons with mental illness. The study’s proposed facial action coding system enabled the interpretation of emotional facial expressions and thus contributed to the knowledge of therapeutic intervention for patients with mental illnesses. This can range from seeing a person engaging in a group in real life to filmed encounters in which facial expressions are recorded under laboratory circumstances after the emotion is elicited experimentally. Using the picture of a filmed face for image processing and capturing precise expression changes (called action units), this technology permits the detection of fundamental emotions throughout time. By utilizing surface electrodes, an electromyography (EMG) approach was created to distinguish the activation of facial muscles as correctly and clearly as feasible. This advancement in technology enabled the detection and independent recording of the actions of the modest visible facial muscles. Automatic face recognition: the quality of commercially available systems for automatic face recognition has significantly increased. The SHORE™ technology, which is the world’s premier face detection system, is the result of years of research and development in the field of intelligent systems. SHORE™ led to the development of a high-performance real-time C++ software library. A significant percentage of people suffer from a nervous system imbalance, which causes paralysis of the patient’s movement and unexpected falls. So, A better understanding of how emotions are regulated and how the dynamics of facial expression of emotion can be explained could lead to a better understanding of the interactive and social consequences of emotional expression deficits in people with mental illness, as well as a therapeutic intervention.

Most patients with any neurological condition have ambulatory disruption at any stage of the disease, which can lead to falls without warning signs, and each patient is unique. As a result, a technique to identify shaky motion is required.

### 4.3. Fall Detection

A thesis topic in [72] is about assessing the real-time gait of a Parkinson’s disease patient to actively respond to unstable motions. They devised a technique to monitor a real-time gait analysis algorithm by wearing SHIMMER wireless sensors on the waist, chest, and hip based on real-world data to see which one is the most suited to identify any gait deviation. This approach is efficient, sensitive to identifying miner deviation, and user-configurable, allowing the user to adjust the sampling rate and threshold settings for motion analysis. Researchers can utilize this technique without having to develop it themselves in their research. The initial sampling rate is set to 100 MHz, and it operates with precalculated threshold values. Accelerometers worn on the chest reveal excessive acceleration during falls, and thus it is best to wear them on the waist. Additionally, as illustrated in the aware gait, if a patient takes steps with vigor, her or his gait may become steadier: the patient still has postural instability and falls following the DBS treatment. As a result, even after surgery, such people may have impaired cognition. Another discovery is that people with this condition may tilt left or right when turning.

Because the suggested approach is sensitive to detecting falls, it may be used objectively to estimate fall risk. The same algorithm, with small tweaks, may be used to identify seizures in different conditions, primarily epileptic seizures, and inform health care personnel in an emergency.

In the medical field, fall detection is a big issue. Elderly folks are more likely than others to fall. People over the age of 65 account for more than half of all injury-related hospitalizations. Commercial fall detection devices are costly and need a monthly subscription to operate. For retirement homes and clinics to establish a smart city powered by AI and IoT, a more inexpensive and customizable solution is required. A reliable fall-detection system would detect a fall and notify the necessary authorities.

In [73], they used edge-computing architecture to monitor real-time patient behavior and detect falls using an LSTM fall detection model. To track human activity, they employed MbientLab’s MetaMotionR wireless wearable sensor devices, which relayed real-time streaming data to an edge device. To analyze the streaming sensor data, we used a laptop as an edge device and built a data analysis pipeline utilizing bespoke APIs from Apache Flink, TensorFlow, and MbientLab. The model is trained by the “MobiAct” dataset, which has been released. The models were shown to be efficient and may be used to analyze appropriate sampling rates, sensor location, and multistream data correction by training them using already public datasets and then improving them. Experiments demonstrated that our architecture properly identified falls 95.8% of the time using real-time sensor data. We found that the optimal location for the sensors is at the waist and that the best data gathering frequency is 50 Hz. We showed that combining many sensors to collect multistream data improves performance.

We would like to expand the framework in the future to include several types of cloud platforms, sensors, and parallel data processing pipelines to provide a system for monitoring patients in clinics, hospitals, and retirement homes. We want to use the MbientLab MetaTracker to construct ML models to identify additional activities and analyze biometrics such as the subject’s heartbeat before and after a fall, sleep pattern, and mobility pattern, as well as track patients’ activity.

### 4.4. Smart Homes in Health Care

**Figure 3:**
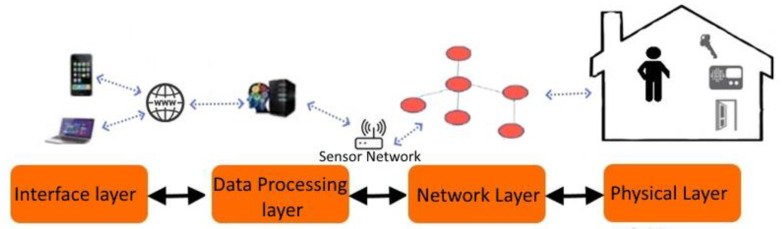
Multilayered architecture of a smart home. The figure was taken from [75].

Many individuals, particularly the elderly and ill, can live alone and keep their freedom and comfort in smart houses. This aim can only be achieved if smart homes monitor all activities in the house and any anomalies are quickly reported to family or nurses. As shown in Figure 3, smart houses feature a multilayered design. The physical layer (environment, objects, and inhabitants), communication layer (wired and wireless sensor network), data processing layer (data storage and machine learning techniques), and interface layer are the four levels (software such as a mobile phone application). Sensors collect data about inhabitants’ activities and the status of the environment, then send it to a server’s data processing layer, where it is evaluated. Users get the results (such as alarms) and interact with the smart home through a software interface. Edge sensors make it easier to monitor various metrics across time, the data is then sent to another device for processing and predictions, this lifts the weight of processing from the sensors to more capable devices, ref. [74] proposes and architecture for smart cameras that allows them to perform high-level inference directly within the sensor without sending the data to another device.

The most common uses of smart homes in health care are automation tasks aiming at activity recognition for a range of objectives, such as activity reminders for Alzheimer’s patients and remote monitoring of people’s health via regulating their vital signs.

#### 4.4.1. Anomaly Detection Using Deep Learning

The authors of [76] used raw outputs from binary sensors, such as motion and door sensors, to train a recurrent network to anticipate which sensor would be turned on/off in the next event, and in this on/off mode, how long it would remain. They then expanded this event into k sequences of successive occurrences using beam search to discover the likely range of forthcoming actions. Several novel approaches for assessing the spatio-temporal sequences’ similarity were used to evaluate the inaccuracy of this prediction, i.e., the distance between these potential sequences and the true string of events. The anomaly scores likelihood can be determined by modeling this inaccuracy as a Gaussian distribution. Abnormal activities will be regarded as input sequences that score higher than a specific threshold. The trials showed that this approach can detect aberrant behaviors with a high level of accuracy.

**Figure 4:**
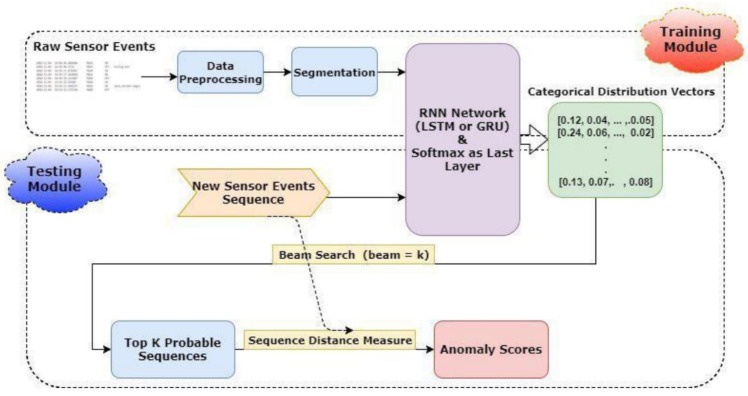
The overall scheme of the proposed method [76].

The suggested method’s general scheme is depicted in Figure 4. The raw sensor events are first preprocessed, which comprises the processes below:

- The SA value is derived by adding the S and A values together.
- SA’s character string has been encoded. This encoding can be done in one of two ways: one-hot encoding or word embedding.
- D is determined by subtracting the current and previous event timestamps.
- The return of time, periodicity, and cycle are all taken into account while converting timestamps.

#### 4.4.2. Anomaly Detection Using Bayesian Networks

A Bayesian network is a representation of a joint probability distribution of a set of random variables with a possible mutual causal relationship. The network consists of nodes representing the random variables, edges between pairs of nodes representing the causal relationship of these nodes, and a conditional probability distribution in each of the nodes. The main objective of the method is to model the posterior conditional probability distribution of outcome (often causal) variable(s) after observing new evidence [77].

The goal of [75] is to identify abnormalities at the proper moment so that harmful situations can be avoided when a person interacts with household products. Its goal is to improve anomaly detection in smart homes by expanding functionality to evaluate raw sensory data and generate suitable guided probabilistic graphical models (Bayesian networks). The idea is to determine the chance of the current sensor turning on and then have the model sound an alarm if the probability falls below a specific threshold. To do this, we create many Bayesian network models of various sizes and analyze them to find the best optimal network with adequate causal links between random variables. The current study is unique in that it uses Bayesian networks to model and train sensory data to detect abnormalities in smart homes. Furthermore, by giving an approach to removing unneeded random variables, identifying the ideal structure of Bayesian networks leads to greater assessment metrics and smaller size. (We look at the first-order Markov property as well as training and evaluating Bayesian networks with various subsets of random variables.)

**Figure 5:**
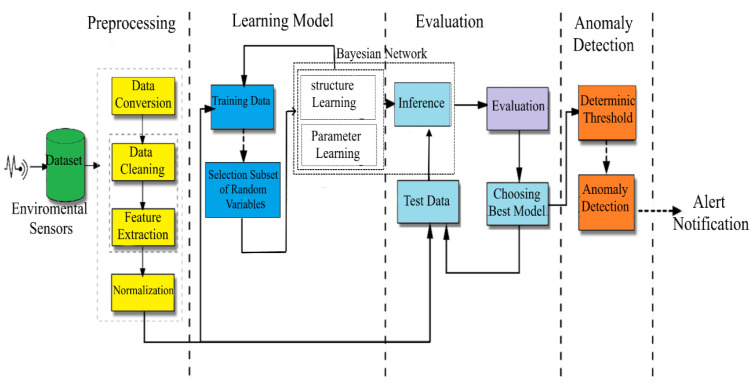
The proposed architecture for anomaly detection in smart homes. The figure has been taken from [75].

We use Bayesian network models to analyze sensory data in smart homes to detect abnormalities and improve occupant safety and health. Pre-processing, model learning, model assessment, and anomaly detection are the four primary steps of the proposed technique Figure 5.

#### 4.4.3. Anomaly Detection Using a Graph-Based Approach

Another approach based on data analysis was presented in [78] for sensor-based smart home settings that have been effectively deployed in the past several years to help elderly persons live more independently. Smart homes are designed to not interfere with inhabitants’ routine activities and to lower the expense of health care connected with their care. Because senior inhabitants are more prone to cognitive health difficulties, analyzing their daily activity using some type of automated tool based on sensor data might offer valuable information about their health state. It is demonstrated that one way to achieve this is to use a graph-based approach to data collected from residents’ activities. It also presents case studies for cognitively impaired participants and discusses how to link these anomalies to the decline in their cognitive abilities, providing clinicians and caregivers with important information about their patients. An unsupervised graph technique has been employed to discover temporal, geographical, and behavioral abnormalities in senior residents’ everyday activities using activity data from smart home sensors. They further hypothesized that these strange actions may indicate a participant’s cognitive deterioration. Data on smart home activities may be created in real-time, as a data stream. They recruited three cognitively challenged people at random for the trial. They would like to change the sample and conduct several trials in the future to see if comparable anomalies may be found. They would also like to look at the graph topology’s resilience to see how much a change in graph topology affects the outcome of anomaly detection. Furthermore, they intend to enlist the help of a doctor as a domain expert to confirm our theory that these abnormalities are true signs of cognitive deterioration (or MCI).

Continuously, it has been planned to expand tests to a real-time data stream in the future. Planned as well is the conversion of real-time sensor logs into graph streams, as well as the search for abnormalities in graph streams, which might allow a real-time health monitoring tool for residents and assist doctors and nurses.

### 4.5. AI for Localizing Neural Posture

The elderly and their struggle to live independently without relying on others was the subject of a study under assessment. The goal of the study [79] was to compare automated learning algorithms used to track their biological functions and motions. Using reference features, the support conveyor algorithm earned the greatest accuracy rate of 95 percent among the eight higher education algorithms evaluated. Long periods of sitting are required in several vocations, which can lead to long-term spine injuries and nervous system illnesses. Some surveys aided in the development of sitting position monitoring systems (SPMS), which use sensors attached to the chair to measure the position of the seated individual. The suggested technique had the disadvantage of requiring too many sensors.

This problem was resolved by designing sitting posture monitoring systems (SPMSs) to help assess the posture of a seated person in real-time and improve sitting posture. To date, SPMS studies have required many sensors mounted on the backrest plate and seat plate of a chair. The present study of [80], therefore, developed a system that measures a total of six sitting postures including the posture that applied a load to the backrest plate, with four load cells mounted only on the seat plate. Various machine learning algorithms were applied to the body weight ratio measured by the developed SPMS to identify the method that most accurately classified the actual sitting posture of the seated person. After classifying the sitting postures with several classifiers, a support vector machine using the radial basis function kernel was used to obtain average and maximum classification rates of 97.20 percent and 97.94 percent, respectively, from nine subjects. The suggested SPMS was able to categorize six sitting postures, including one with backrest loading, and demonstrated that the sitting posture can be classified even when the number of sensors is reduced.

Another posture can we share here is for patients who are in the hospital for an extended period, pressure ulcer prevention is critical. To arrange posture modification for patients, a human body lying posture (HBLP) monitoring system is required. The traditional technique of HBLP monitoring, video surveillance, has several drawbacks, including subject privacy and field-of-view occlusion. With no sensors or wires attached to the body and no limits imposed on the subject, the paper [81] presented an autonomous technique for identifying the four state-of-the-art HBLPs in healthy adult subjects: supine, prone, left, and right lateral. Experiments using a collection of textile pressure sensors implanted in a cover put beneath the bedsheet were done on 12 healthy persons (ages 27.35 5.39 years). A supervised artificial neural network classification model was given a histogram of directed gradients and local binary patterns. Scaled conjugate gradient back-propagation was used to train the model. To evaluate the classification’s generalization performance, nested cross-validation with an exhaustive outer validation loop was used. Intriguingly, a high testing prediction accuracy of 97.9% was found, with a Cohen’s kappa coefficient of 97.2 percent. In contrast to most previous similar studies, the classification successfully separated prone and supine postures. They discovered that combining body weight distribution information with shape and edge information improves classification performance and the capacity to distinguish between supine and prone positions. The findings are encouraging in terms of unobtrusively monitoring posture for ulcer prevention. Sleep studies, post-surgical treatments, and other applications that need HBLP identification can all benefit from the approach.

In patients with myopathy, peripheral neuropathy, plexopathy, or cervical/lumbar radiculopathy, needle electromyography (EMG) is utilized to diagnose a neurological injury. Because needle EMG is such an intrusive exam, it is critical to keep the discomfort to a minimum during inspections. The Electrodiagnosis Support System (ESS), a clinical decision support system specialized for upper-limb neurological damage diagnosis, has been described in the work [82]. ESS can help users through the diagnostic process and make the best option for eliminating unwanted examinations, as well as serve as a teaching tool for medical students. Users may input the results of needle EMG testing and get diagnosis findings using ESS’s graphical user interface, which depicts the neurological anatomy of the upper limb. We used the diagnostic data of 133 real patients to test the system’s accuracy.

### 4.6. AI for Monitoring Patients

In the recent decade, automated patient monitoring in hospital settings has received considerable attention. An essential issue is mental patient behavior analysis, where good monitoring can reduce the risk of injury to hospital workers, property, and the patients themselves.

**Figure 6:**
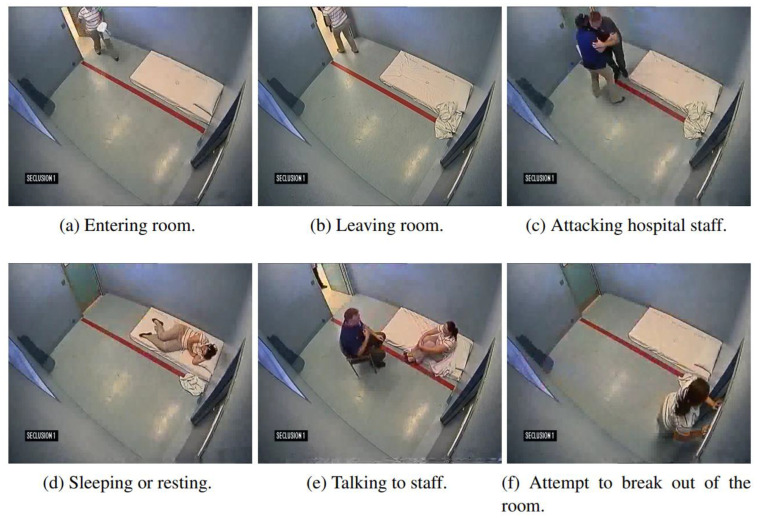
Example of activities to be detected. These images have been taken from [83].

For this task, a computer vision system for monitoring patients was created in safe rooms in hospitals to evaluate their movements and determine the danger of hazardous behavior by extracting visual data from cameras mounted in their rooms. To identify harmful behavior, the proposed technique leverages statistics of optical flow vectors collected from patient motions. Additionally, the approach uses foreground segmentation and blob tracking to extract the shape and temporal properties of blobs such as arriving and leaving the room, sleeping, fighting, conversing, and attempting to escape as shown in Figure 6. Preliminary findings suggest that the technology might be used in a real hospital setting to help avoid harm to patients and employees. A more advanced classification framework for merging the characteristics might be used to increase the system performance and attain a practically low error rate.

Intelligent sensing sensors and wireless communication networks are used in smart health care equipment and applications. The goal of this integration is to improve patient monitoring and make minor illness detection easier.

**Figure 7:**
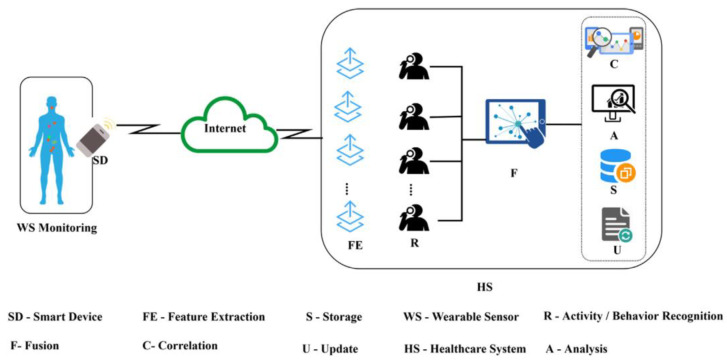
WS to the health care system has been taken from [84].

The study conducted by [84] presents a multilevel decision system (MDS) for recognizing and monitoring patient behavior based on sensed data. Wearable sensing devices are implanted in the body to detect physiological changes at set intervals. The data collected by these sensors is utilized by the health care system (HS) to diagnose and predict illnesses. In this suggested MDS, there are two layers of decision-making: the first is aimed to speed up the data collection and fusion process. Data correlation is used to detect certain behaviors during the second-level decision process. Inter-level optimization reduces errors by fusing multi-window sensor data, allowing for correlation. This optimization acts as a bridge between the first and second decision-making stages. The wearable sensor and health care system are depicted as part of the decision-making process. Using multi-window fusion decision-making, the health care system (HS) in Figure 7 performs activity/behavior extraction, data fusion, and feature extraction. It has data streaming characteristics that make it easier to make decisions, even with nonlinear sensor results. Storage, updating, analysis, and correlation of sensor data are carried out in the second decision-making phase. The data from the body-worn wearable sensors is compiled on a smart handheld device (e.g., cellphones, digital gadgets) and sent to the HS over the Internet.

The patient’s behavior and the type of the ailment were recognized based on this information for use in future diagnosis and prediction. MDS also uses flexible information analysis to match patient behavioral analysis and come up with improved recommendations. MDS’s dependability is demonstrated by experimental analysis, which improves the true positive rate, F-measure score, and accuracy fusion latency.

### 4.7. AI and Patient’s Lower Limb Movement

The qualitative and quantitative study of climbing, running, and human walking is referred to as HUMAN lower limb motion analysis. It is based on kinematic notions as well as human anatomy and physiology, and it is frequently used in augmented virtual reality, foot navigation, and medical rehabilitation, among other applications [85].

#### 4.7.1. Evaluation of Paraplegics’ Legged Mobility

The inability to walk and stand is one of the most important disabilities caused by paraplegia. Along with a reduction in movement. This research [86] examined a lower limb exoskeleton for paraplegics who require leg movement. The research offers a single-subject case study on a patient with a T10 motor and sensory complete damage, comparing legged movement using an exoskeleton versus locomotion using knee–ankle–foot orthoses (KAFOs). The timed up-and-go test, the Ten-Meter Walk Test (10 MWT), and the six-minute walk test (6 MWT) are used to measure the subject’s capacity to stand, walk, turn, and sit. The Physiological Cost Index was used to determine the level of effort associated with each evaluation tool. Results indicate that the subject was able to perform the respective assessment instruments 25%, 70%, and 80% faster with the exoskeleton relative to the KAFOs for the timed up-and-go test, the 10 MWT, and the 6 MWT, respectively. Measurements of exertion indicate that the exoskeleton requires 1.6, 5.2, and 3.2 times less exertion than the KAFOs for each respective assessment instrument. The results indicate that the enhancement in speed and reduction in exertion is more significant during walking than during gait transitions.

#### 4.7.2. Estimating Clinically of Strokes in Gait Speed Changing

In persons who have had a stroke, gait speed is routinely used to determine walking capacity. It is unclear how much of a difference in gait speed corresponds to a significant change in walking capacity. The goal of the study [87] was to quantify clinically significant changes in gait speed using two distinct anchors for “significant”: Perceptions of progress in walking capacity among stroke survivors and physical therapists. After a first-time stroke, the participants received outpatient physical treatment (mean 56 days post-stroke). At admission and discharge, self-selected walking speed was assessed. On a 15-point ordinal global rating of change (GROC) scale, subjects and their physical therapists scored their perceived change in walking ability after discharge. Using receiver operating characteristic curves and the participants’ and physical therapists’ GROC as anchors, estimated relevant change values for gait speed were determined. All the subjects’ initial gait speeds were 0.56 (0.22) m/s on average. Depending on the anchor, the assessed significant change in gait speed was between 0.175 m/s (participants felt the change in walking ability) and 0.190 m/s (physical therapists perceived change in walking ability). Individuals who increase their gait speed by 0.175 m/s or more during the subacute period of rehabilitation are more likely to have a considerable improvement in walking ability. Clinicians and researchers can utilize the estimated clinically relevant change value of 0.175 m/s to establish objectives and analyze the change in individual patients, as well as to compare important changes between groups.

#### 4.7.3. Measuring Parkinson’s Gait Quality

Wearable sensors that monitor gait quality in daily activities have the potential to improve medical evaluation of Parkinson’s disease (PD). Four gait partitioning strategies were examined in the work [88], two based on machine learning and two based on the thresholds approach, all using the four-phase model. During walking tasks, the approaches were evaluated on 26 PD patients in both ON and OFF levodopa circumstances, as well as 11 healthy volunteers. All the participants wore inertial sensors on their feet. The reference time sequence of gait phases was assessed using force resistive sensors. To determine the accuracy of gait phase estimation was using the goodness index (G). For gait quality evaluation, a new synthetic index termed the gait phase quality index (GPQI) was developed. The results indicated that three of the examined techniques had optimal performance (G 0.25) and one threshold approach had acceptable performance (0.25 G 0.70). The GPQI was shown to be considerably higher in PD patients than in healthy controls, with a modest connection with clinical scale scores. Furthermore, GPQI was shown to be greater in the OFF state than in the ON state in individuals with significant gait impairment. Our findings show that real-time gait segmentation based on wearable sensors may be used to assess gait quality in people with Parkinson’s disease.

### 4.8. Remark

Recent advancements in low-cost smart home devices and wireless sensor technology have resulted in an explosion of small, portable sensors that can measure body motion rapidly and precisely. Movement-tracking technologies that are both practical and beneficial are now available. Therapists need to be aware of the possible benefits and drawbacks of such new technology. As said in [89], therapists may be able to undertake telerehabilitation in the future using body-worn sensors to assess compliance with home exercise regimens and the quality of their natural movement in the community. Therapists want technology tools that are simple to use and give actionable data and reports to their patients and referring doctors. Therapists should search for systems that have been evaluated in terms of gold standard accuracy as well as clinically relevant outcomes such as fall risk and impairment severity.

## 5. AI and COVID-19

### 5.1. Overview

The medical sector is seeking innovative tools to monitor and manage the spread of COVID-19 in this global health disaster. Artificial intelligence (AI), the Internet of Things (IoT), big data, and machine learning are technologies that can readily track the transmission of this virus, identify high-risk individuals, anticipate new illnesses, and aid in real-time infection management. These technologies might also forecast mortality risk by thoroughly evaluating patients’ historical data.

The study by [90] examined the role of artificial intelligence (AI) as a critical tool for analyzing, preparing for, and combating COVID-19 (Coronavirus) and other pandemics. AI can aid in the fight against the virus by providing population screening, medical assistance, notification, and infection control recommendations. As an evidence-based medical tool, this technology has the potential to enhance the COVID-19 patient’s planning, treatment, and reported outcomes.

Artificial Intelligence (AI) is an emerging and promising technology for detecting early coronavirus infections and monitoring the state of affected individuals. It can monitor the COVID-19 outbreak at many scales, including medical, molecular, and epidemiological applications. It is also beneficial to aid viral research by evaluating the existing data. Artificial intelligence can aid in the creation of effective treatment regimens, preventative initiatives, and medication and vaccine development.

**Figure 8:**
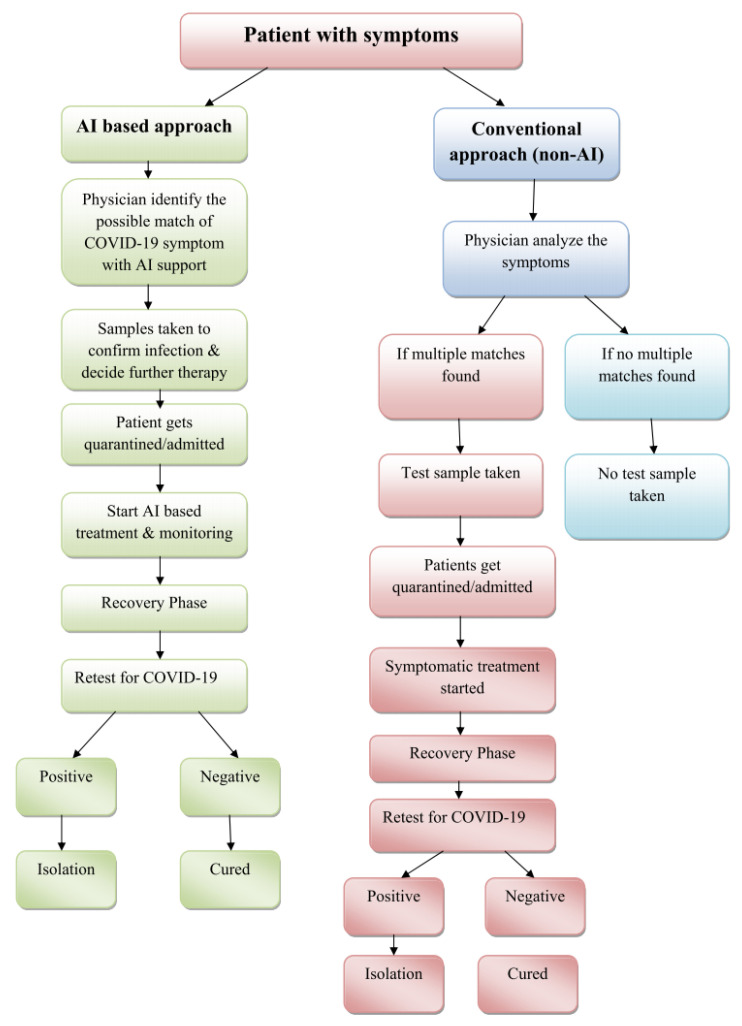
The general procedure of AI and non-AI-based applications help general physicians to identify the COVID-19 symptoms. This figure has been taken from [90].

The basic approach of AI and non-AI-based programs that assist general physicians in identifying COVID-19 symptoms is shown in Figure 8. The flow diagram below illustrates and contrasts the flow of minimum non-AI versus AI-based therapy. The flow diagram below demonstrates how AI is used in key aspects of high-accuracy therapy, reducing the complexity and time required. With the AI application, the physician is not only focused on the patient’s therapy, but also illness control. AI is used to analyze major symptoms and test results with the highest level of accuracy. It also demonstrates that it minimizes the overall number of steps in the entire process, making it more readily available in nature.

### 5.2. AI Training Techniques

The medical field makes use of two different paradigms when it comes to AI, supervised where the data is labeled and the model learns to map features to an outcome, the outcome is known beforehand, making it easier to score the model and track its performance. The other technique utilized is unsupervised learning, unlike supervised learning, unsupervised learning uses unlabeled and unstructured data that is fed to a model and giving it the opportunity to learn and extract useful information from the data as it sees fit, these techniques are utilized for various other tasks such as early warning systems and faster cure discovery.

#### 5.2.1. Supervised Learning

Supervised Learning is one of the most often used techniques in the health care system, and it is well-established. This learning approach makes use of labeled data X with a provided target Y to learn how to predict the correct value of Y given input X.

Supervised learning can help provide a solid foundation for COVID-19 planned observation and forecasting. A neurological system might also be developed to extract the visual features of this disease, which would aid in the proper diagnosis and treatment of those who are affected. An Xception, depth-wise based CNN technique for convolution distinct layers has been presented in [91]. Two convolution layers are at the top, followed by a related layer, four convolution layers, and depth-wise divisible convolution layers. In research from [80], it was used to identify bed positions using a variety of bed pressure sensors. It can be a beneficial weapon in battling the COVID-19 because of its capabilities and high-efficiency outcome.

#### 5.2.2. Unsupervised Learning

Instead of using verified data as in the previous learning strategy, this learning technique employs names without information signals. This method is widely used to discover covered structures in data and divide them into small groups. Its primary purpose is to present and construct a clear differentiating proof. This is a potential type of estimation for meeting the general AI requirement, although it lags far behind the previously stated learning approach. The autoencoder [92] and K-means [93] are the most well-known unsupervised techniques. The quirk acknowledgment [94] is one of the most widely seen duties of this learning strategy in the medical field. The affiliation data will begin with comparable scattering; if there is any form of interference, as an exception, this data point can be hailed or observed without difficulty. There are many solutions that are relatively cheap and allow deploying AI models in a fast way such as Nividia’s Jetson nano kit, a Raspberry Pi, or Google’s coral, for example. Therefore, this concept may be used to CT scan pictures as well as other medical applications, such as for COVID-19.

**Figure 9:**
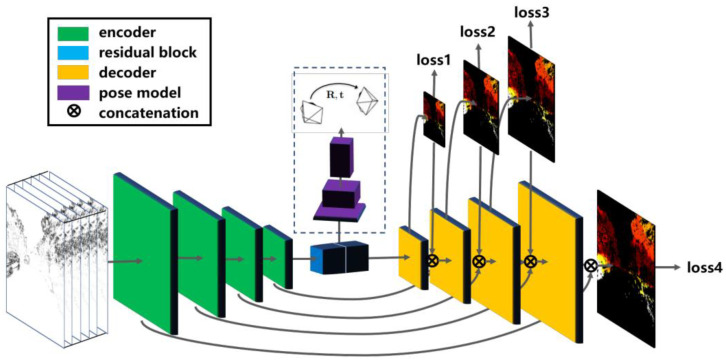
Network architecture for both the optical flow and egomotion and depth networks. This figure has been taken from [95].

The author proposed a new framework for this learning model for opportunistic cameras that record moving data from a stream [95]. The neural system is then used to predict how the event broadcast will move. This movement is used to attempt and remove any movement that is concealed in the streaming images. To further explain this concept, we depict the applied learning approach in Figure 9, where the training and testing attributes collected from the patient are denoted by the symbol X. In this case, accuracy is not a priority; instead, the approach’s purpose is to uncover any interesting examples that may be found among the available data. Furthermore, additional information can be used to corroborate or disprove the samples it detects.

### 5.3. Real World Use Cases

The contribution of AI to the fight against COVID-19 is discussed in this paper [96], as well as the present restrictions on these efforts. (i) early warnings and alerts, (ii) prediction and tracking, (iii) data dashboards, (iv) diagnosis, (v) cures and treatment, and (vi) health care workers’ workloads are being reduced are six areas where AI may help in the battle against COVID-19. The conclusion is that AI has yet to influence COVID-19. Its utilization is restricted by a lack of data as well as an abundance of data. To overcome these limitations, a careful balance between data privacy and public health, as well as rigorous human-AI interaction, will be required. These are unlikely to be addressed in time to be of much use during the current pandemic. Meanwhile, a large-scale collection of diagnostic data on who is infectious will be required to save lives, train AI, and reduce economic losses. In the works [93], Different AI techniques are utilized for COVID-19 detection, these techniques despite their major differences provide admirable results that helped in making it easier and faster to detect the spread of COVID-19, the discussed techniques will be further explained in detail below.

#### 5.3.1. Early Warnings and Alerts

AI can swiftly identify unusual symptoms and other red flags, alerting patients and health care providers [97]. It aids in cost-effective decision making by allowing for speedier decision making. Through relevant algorithms, it aids in the development of a novel diagnosis and management strategy for COVID-19 patients. With the use of medical imaging technologies such as computed tomography (CT) and magnetic resonance imaging (MRI) scans of human body parts, AI can assist in the identification of infected patients.

For example, BlueDot 4, a Canadian AI model, demonstrates how a low-cost AI tool (BlueDot was supported by a startup investment of roughly US$ 9 million) may outperform humans at detecting infectious disease epidemics as shown in [98]. According to reports, BlueDot foresaw the epidemic at the end of 2019, giving a warning to its clients on 31 December 2019, one day before the World Health Organization announced so on 9 January 2020. In [99], a group of academics worked with BlueDot and compiled a list of the top 20 destinations for travelers flying from Wuhan after the epidemic. They cautioned that these cities might be at the front of the global spread of the disease.

While BlueDot is unquestionably a strong tool, much of the press around it has been exaggerated and undervalues the contribution of human scientists. First, while BlueDot raised an alarm on 31 December 2019, another AI-based model at Boston Children’s Hospital (USA), reading the HealthMap in [100], raised a warning on 30 December 2019.

#### 5.3.2. Prediction and Tracking

AI may be used to track and forecast the spread of COVID-19 over time and space. A neural network may be built to extract visual aspects of this condition, which would aid inadequate monitoring [101]. It has the potential to offer daily information on patients as well as remedies to be implemented in the COVID-19 pandemic.

For example, during a previous pandemic in 2015, a dynamic neural network to anticipate the spread of the Zika virus was constructed. Models such as these, on the other hand, will need to be retrained using data from the COVID-19 pandemic. Various projects are underway to collect training data from the present epidemic, as detailed below.

Various issues plague accurate pandemic predictions; see, for example, [102]. This includes a dearth of historical data on which to train the AI, panic behavior that causes “noise” on social media, and the fact that COVID-19 infections have different features than prior pandemics. Not only is there a paucity of historical data, but there are also issues with employing “big data”, such as information gleaned from social media. The risks of big data and AI in the context of infectious illnesses, as demonstrated by Google Flu Trends’ notable failure, remain valid. “Big data hubris and algorithm dynamics”, as [103] put it. For example, as the virus spreads and the quantity of social media traffic around it grows, so does the amount of noise that must be filtered out before important patterns can be recognized.

AI estimates of COVID-19 spread are not yet particularly accurate or dependable because of a lack of data, big data hubris, algorithmic dynamics, and loud social media.

As a result, most tracking and forecasting models do not employ AI technologies. Instead, most forecasters choose well-established epidemiological models, often known as SIR models, which stand for susceptible, infected, and removed populations in a certain area. The Institute for the Future of Humanity at Oxford University, for example, uses the GLEAMviz epidemiological model to anticipate the virus’s spread, looking in [104].

An Epidemic Tracker model of illness propagation is available from Metabiota Looking forward to a San Francisco-based startup [105]. In a YouTube video, watching, Crawford, an Oxford University mathematician, gives a simple and concise explanation of SIR models [106].

The Robert Koch Institute in Berlin employs an epidemiological SIR model that incorporates government containment measures including quarantines, lockdowns, and social distancing, the model is explained here [107]. In [108] recently, it has been pre-published and made it accessible in R format and enhanced the SIR model that takes into consideration public health interventions against the pandemic and uses data from China.

The Robert Kock Institute’s model has already been utilized in the instance of China to show that containment can be effective in slowing the spread to less than exponential rates [107].

#### 5.3.3. Data Dashboards

**Figure 10:**
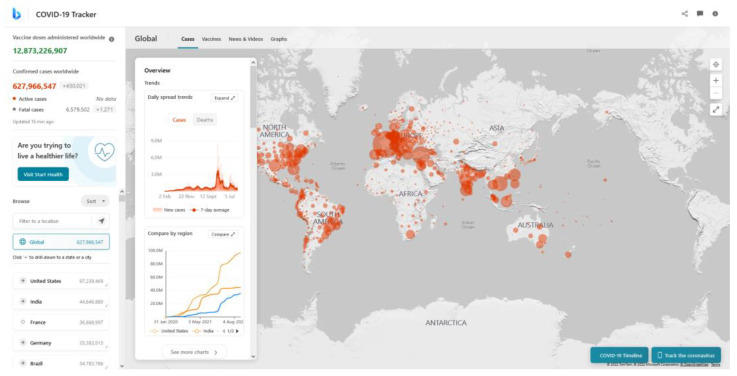
Microsoft Bing’s COVID-19 Tracker, note(s): Screenshot of Bing’s COVID-19 Tracker, 9 February 2022.

COVID-19 tracking and forecasting has spawned a cottage industry of data dashboards for visualizing the actual and predicted spread. The MIT Technology Review [109] has ranked these dashboards for tracking and forecasting. HealthMap, UpCode, Thebaselab, NextStrain, the BBC, Johns Hopkins’ CSSE, and the New York Times have the best dashboards, according to them. Microsoft Bing’s COVID-19 Tracker is another important dashboard see Figure 10.

While these dashboards provide an increasing number of cites, and a global overview, have their dashboards in place. For example, South Africa established the COVID-19 ZA South Africa Dashboard which is maintained by the University of Pretoria’s Data Science for Social Impact Research Group [110].

Tableau has produced a COVID-19 data hub with a COVID-19 Starter Workbook to help with the creation of data visualizations and dashboards for the epidemic [111].

#### 5.3.4. Diagnosis

COVID-19 diagnosis that is quick and accurate can save lives, prevent disease transmission, and produce data for AI models to learn from. In this case, AI might be helpful, especially when establishing a diagnosis based on chest radiography pictures. In a recent assessment of artificial intelligence applications versus coronaviruses, studies have demonstrated that AI can be as accurate as humans, save radiologists’ time, and provide a diagnosis faster and cheaper than normal COVID-19 tests [112].

For COVID-19, AI can save radiologists time and help them diagnose the disease faster and more affordably than current diagnostics. X-rays and computed tomography (CT) scans are both options. A lesson on how to diagnose COVID-19 utilizing X-ray pictures using Deep Learning has been provided in [113]. COVID-19 tests are “in low supply and costly”, he points out, but “all hospitals have X-ray machines”. A method for scanning CT scans with mobile phones has been presented in [114].

In this context, several projects are in the works. COVID-Net, has been created by [115], is a deep convolutional neural network (see, for example, [116]) that can diagnose coronavirus from chest X-RAY pictures. It was trained using data from roughly 13,000 individuals with diverse lung diseases, including COVID-19, from an open repository. However, as the authors point out, it is “far from a production-ready solution”, and they urge the scientific community to continue working on it, especially to “increase sensitivity” (Ibid, p.6). A Deep Learning model has been presented in [117] to diagnose COVID-19 from CT scans (which has not yet been peer-reviewed), concluding that “The deep learning model showed comparable performance with an expert radiologist, and greatly improve the efficiency of radiologists in clinical practice. It holds great potential to relieve the pressure off frontline radiologists, improve early diagnosis, isolation, and treatment, and thus contribute to the control of the epidemic”. (Ibid, p.1).

Researchers from the Dutch University of Delft, for example, developed an AI model for detecting coronavirus from X-rays at the end of March 2020. On their website available in [111], this model, dubbed CAD4COVID, is touted as an “artificial intelligence program that triages COVID-19 suspicions on chest X-ray pictures”. It is based on prior AI models for TB diagnosis created by the institution.

Although it has been claimed that a handful of Chinese hospitals have installed “AI-assisted” radiology technologies for example see the report in [118], the promise has yet to be realized. Radiologists in other countries have voiced worry that there is not enough data to train AI models, that most COVID-19 pictures are from Chinese hospitals and may be biased, and that utilizing CT scans and X-rays might contaminate equipment and spread the disease further.

Finally, once one has been diagnosed with the disease, the question of whether and how severely that one will be affected arises. COVID-19 does not always need rigorous treatment. Being able to predict who will be impacted more severely can aid in the targeting of assistance and the allocation and utilization of medical resources. Only 29 patients at Tongji Hospital in Wuhan, China, the authoers of [119] used the data of thus patients to develop a prognostic prediction algorithm to forecast the mortality risk of a person who has been infected. Howevere, the authores in [120] have offered an AI that can predict with 80% accuracy who would suffer acute respiratory distress syndrome after contracting COVID-19 (ARDS).

#### 5.3.5. Faster Cure Discovery

Long before the coronavirus epidemic, AI was praised for its ability to aid in the development of novel drugs see for example [121]. In the instance of coronavirus, several types of research institutes and data centers have already said that AI would be used to find therapies and a vaccine for the virus. The goal is that artificial intelligence will speed up both the discovery and repurposing of current medications. By assessing the existing data on COVID-19, AI is employed for medication research. It may be used to design and develop medication delivery systems. This technique is utilized to speed up drug testing in real-time when normal testing takes a long time, and so helps to considerably speed up this procedure, which would be impossible for a human to do. For example, Google’s DeepMind, which is best known for its AlphaGo game-playing algorithm, AI has been used in [122] to anticipate the structure of viral proteins, which might aid in the development of novel treatments. DeepMind, on the other hand, makes it explicit on its website associated with COVID-19, 2020) that “we emphasize that these structure predictions have not been experimentally verified…we can’t be certain of the accuracy of the structures we are providing”.

#### 5.3.6. Repurposing Existing Drugs

Beck et, al. [123] provides findings from a study that used Machine Learning to determine if an existing medicine, atazanavir, may be repurposed for coronavirus treatment. And, in collaboration with Benevolent AI, a UK AI business, [101] discovered Baricitinib, a drug used to treat myelofibrosis and rheumatoid arthritis, as a viable COVID-19 therapy. AI can assist in the discovery of effective medications to treat coronavirus patients. It has evolved into a useful tool for developing diagnostic tests and vaccines [124]. In research from [125], AI aids in the creation of vaccines and therapies at a much faster rate than before, as well as clinical trials during vaccine development.

### 5.4. AI and Health Care Workers’ Workloads Reduction

Health care workers are overworked because of a sudden and significant increase in the number of patients during the COVID-19 epidemic. In this case, artificial intelligence (AI) is employed to lessen the burden on health care staff. Hence, in research from [85] utilizing the classification of confirmed instances of coronavirus (new version of COVID-19) as one of the pandemic illnesses, a severe problem in the sustainable development process was studied. As a result, binary classification modeling was employed as one of the artificial intelligence ways using the group method of data handling (GMDH) kind of neural network. The suggested model was built using the Hubei province of China as a case study, with certain significant characteristics such as minimum, average, and maximum city density, relative humidity, daily temperature, and wind speed as input datasets, and the number of verified cases as output dataset for 30 days.

The suggested binary classification model outperforms the competition in terms of predicting confirmed instances. In addition, regression analysis was performed, and the trend of confirmed cases was compared to daily weather parameter changes (humidity, average temperature, and wind).

The relative maximum day temperature and humidity had the greatest influence on the verified cases, according to the findings. The confirmed cases were impacted positively by the relative humidity in the primary case study, which averaged 77.9%, and adversely by the highest daily temperature, which averaged 15.4 °C.

Offering the greatest training to students and clinicians on this emerging illness by utilizing digital techniques and decision science [126]. AI can improve future patient care and handle more possible difficulties, reducing doctors’ burden.

### 5.5. Remark

From an epidemiological, diagnostic, and pharmacological standpoint, AI has yet to play a substantial part in the fight against coronavirus. Its application is limited by a shortage of data, outlier data, and an abundance of noise. It is vital to create unbiased time series data for Artificial intelligence training. While the expanding number of worldwide activities in this area is promising, more diagnostic testing is required. Not just for supplying training data for AI models, but also better controlling the epidemic and lowering the cost in terms of human lives and economic harm.

Data is crucial in determining if AI can be used to combat future diseases and pandemics. As in [96], it has been previously stated that the risk is public health reasons will override data privacy concerns. Long after the epidemic has passed, governments may choose to continue the unparalleled surveillance of their population. As a result, worries regarding data privacy are reasonable.

## 6. Significance of the Study (Body Language Symptoms for COVID-19)

Communication is one of the most crucial skills a physician should have, according to patient surveys. However, communication encompasses more than just what is spoken. From the time a patient first visits a physician, his or her nonverbal communication, or body language, determines the course of therapy. Bodily language encompasses all nonverbal forms of communication, including posture, facial expression, and body movements. Being aware of such habits can help doctors gain more access to their patients. Patient involvement, compliance, and the result can all be influenced by effective nonverbal communication [127].

Pandemic and epidemic illnesses are a worldwide threat that might kill millions of people. Doctors have limited abilities to recognize and treat victims. Human and technological resources are still in short supply when it comes to epidemic and pandemic conditions. To better the treatment process and when the patient is unable to travel to the treatment location, remote diagnosis is necessary, and the patient’s status should be automatically examined. Altering facial wrinkles, movements of the eyes and eyebrows, some protrusion of the nose, changing the lips, and the appearance of certain motions of the hands, shoulders, chest, head, and other areas of the body are all characteristics of pandemic and epidemic illnesses. Artificial intelligence technology has shown promise in understanding these motions and cues in some cases. As a result, the concept of allocating body language to identifying epidemic diseases in patients early, treating them early, and assisting doctors in recognizing them arose owing to the speed with which they spread and people died. It should be emphasized that the COVID-19 sickness, which horrified the entire world and revolutionized the world’s life, was the major and crucial motivator for the idea of this study after we studied the body language analysis research in health care and defined the automatic recognition frame using artificial intelligence to recognize various body language elements.

As researchers in information technology and computer science, we must contribute to discussing an automatic gesture recognition model that helps better identify the external symptoms of epidemic and pandemic diseases for helping mankind.

## 7. Conclusions

In this paper, we reviewed the recent literature analyzing patients’ body language using deep learning techniques. Since most of this research is ongoing, we focused on the body language analysis research in health care. In such recent works, most of the research in health care has been considered to define the automatic recognition frame using artificial intelligence to recognize various body language elements. It will be interesting to discuss an automatic gesture recognition model that helps better identify the external symptoms of epidemic and pandemic diseases.

The body language analysis of patients using artificial intelligence for identifying the external symptoms of epidemic and pandemic diseases is a motivating issue for future research to improve the process of treatment, including for when the patient is inaccessible to the place of the treatment, remote diagnosis is required, and the patient’s condition should be analyzed automatically.

## Data Availability

Not Applicable.
